# PANGAEA - Data Publisher for Earth & Environmental Science

**DOI:** 10.1038/s41597-023-02269-x

**Published:** 2023-06-02

**Authors:** Janine Felden, Lars Möller, Uwe Schindler, Robert Huber, Stefanie Schumacher, Roland Koppe, Michael Diepenbroek, Frank Oliver Glöckner

**Affiliations:** 1 Alfred Wegener Institute, Helmholtz Center for Polar- and Marine Research, Bremerhaven, Germany; 2 MARUM, Center for Marine Environmental Sciences, University of Bremen, Bremen, Germany; 3 German Federation for Biological Data (GFBio) e.V., Bremen, Germany

**Keywords:** Environmental sciences, Planetary science

## Abstract

The information system PANGAEA provides targeted support for research data management as well as long-term data archiving and publication. PANGAEA is operated as an open access library for archiving, publishing, and distributing georeferenced data from earth and environmental sciences. It focuses on observational and experimental data. Citability, comprehensive metadata descriptions, interoperability of data and metadata, a high degree of structural and semantic harmonization of the data inventory as well as the commitment of the hosting institutions ensures the long-term usability of archived data. PANGAEA is a pioneer of FAIR and open data infrastructures to enable data intensive science and an integral component of national and international science and technology activities. This paper provides an overview of the recent organisational, structural, and technological advancements in developing and operating the information system.

## Introduction

PANGAEA - Data Publisher for Earth & Environmental Sciences (www.pangaea.de) has a 30-year history as an open-access library for archiving, publishing, and disseminating georeferenced data from the earth, environmental and biodiversity sciences. It is operated as a joint facility of the Alfred Wegener Institute, Helmholtz Centre for Polar- and Marine Research (AWI) and the Center for Marine Environmental Sciences (MARUM) at the University of Bremen. PANGAEA emerged from the SEDAT/SEDAN database established 1987 built to structure information about sediment cores and promote “Sediment Data Analysis”. Seven years later the foundation of the “information system for long-term archiving and publication of data from earth and environmental science” was initiated followed by the renaming to PANGAEA as the “PAleoNetwork for GeologicAl and EnvironmentAl data. In 2010 this was finally changed to its current name and more generic objective: PANGAEA “Data Publisher for Earth & Environmental Science”. Today PANGAEA holds a mandate from the World Meteorological Organization (WMO, https://public.wmo.int/) to host the World Radiation Monitoring Center (WRMC, https://bsrn.awi.de/). It is accredited as a World Data Center by the International Council for Science (ICS, https://worlddatasystem.org/) since 2001 and has been certified as a trustworthy long-term data archive by Core Trust Seal (https://www.coretrustseal.org/) since 2019.

Technically PANGAEA started as a file-based data system which was changed in 1994 for the first relational database to allow harmonization of the (meta)data. In 1998 PANGAEAs first webpage went online which was refactored for enhanced usability in 2016. Since 2004 each published dataset can be referenced by a universally unique Digital Object Identifier (DOI). Recent highlights were the introduction of the ticket system in 2011 to facilitate and document the interactions with the data submitters, a Twitter account (https://twitter.com/PANGAEAdataPubl) in 2019 and the rating of the datasets via social networks including altmetric (www.altmetric.com). In 2021 PANGAEA’s submission system was refactored for usability and finally in 2022 usage statistics for each dataset were introduced.

**Fig. 1:**
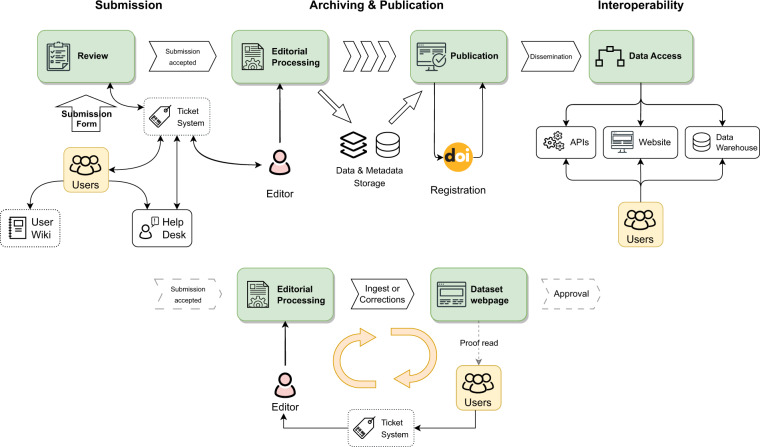
Overview of PANGAEAs workflow from data submission to dissemination.

This publication provides an overview of the current status of PANGAEA, following the data flow from submission to publication and dissemination in the PANGAEA information system. A general outline of the workflow and interactions between submitters and the PANGAEA team is given in Fig. 1.

In the submission process all data and metadata are compiled in close collaboration with the scientists and domain expert data editors following an OAIS^1^ compliant ingest procedure. A broad spectrum of ISO 19115 compliant contextual information (metadata), explaining the where, how, when, and why of a measurement is requested for each dataset. Additionally, the methods and devices used for sampling and analysis of the data as well as references to related literature and external resources are gathered.

In the archiving and publication process both data and metadata are structurally harmonized and checked for completeness and plausibility. Semantic interoperability is ensured through a mapping between a number of metadata properties and standardised terminologies according to international protocols and standards^2^. This harmonization and standardization promotes not only machine readability and further processability, but also a high degree of reusability of the data stock, in compliance with the FAIR data principles^3^. All published datasets carry a licence information (CC0 or CC-BY) and are equipped with a unique DOI to facilitate proper citation of the dataset. Data citation is aligned with the principles and recommendations of ESIP^4^ and FORCE11^5^ as well as the guidelines and standards of DataCite (https://datacite.org/), facilitating crosslinking to manuscripts and data.

Interoperability and findability of data and metadata is enforced by PANGAEAs versatile metadata framework, semantic layers and tools for programmatic data access. This allows effective dissemination of data and metadata to major internet search-engine registries, library catalogues, data portals, and other service providers. The integration of multiple internationally endorsed persistent identifier systems (PIDs) at PANGAEA complement the range of interconnectivity options for individual scientific resources and information. The successful cooperation between PANGAEA and publishers enables the cross-referencing of scientific publications and datasets. PANGAEA is the recommended data repository of numerous international scientific journals.

As of May 2023, PANGAEA provides access to over 419,000 datasets containing over 25 billion individual measurements and observations collected by thousands of individual researchers through more than 785 national and international projects.

## Results

### Operation

PANGAEA is long-term operated by its hosting institutions, which provides the funding for its basic operations. Additional funds for data management and technical innovations are received from third-party funding for scientific projects (international, EU, national; https://www.pangaea.de/projects/).

PANGAEA’s Editorial-team is responsible for the review and quality assessment of data submissions, data management and curation. It meets bi-weekly to exchange knowledge, resolve issues, and discuss the status and development of the system and editorial workflows. Additionally, the team meets several times a week in smaller groups to harmonize specific issues in curation. New editorial staff is trained by senior editors and experienced tutors over a period of several months. There are regular training sessions for Standard Operating Procedures (SOPs) and to keep editors up to date. New staff is recruited mostly from the scientific environment related to earth, environmental and biological sciences.

PANGAEA’s Tech-team is responsible for the maintenance and further development of the technical infrastructure. It meets several times a week to monitor the operation of the systems and plan new developments. It uses the agile programming (scrum) framework and defines regular two-week sprints. Furthermore, members of the Editorial- and Tech-teams hold regular joint meetings to ensure alignment of developments and resolve any upcoming issue.

To scale with the increasing amount of data submissions and make use of the local expertise of its large user community, PANGAEA follows a front-office/back-office model. In particular, PANGAEA offers its data archiving and publishing backbone (the back-office) for usage by other institutes and workgroups (the front-office). To become a PANGAEA front-office affiliate a cooperation agreement must be signed which defines the rights and obligations of the contractors. All data stewards and editors working in the front-office are trained and continuously mentored by the Editorial-team to assure that PANGAEAs editorial standards are met.

### Submission

PANGAEA accepts data from most fields of earth, environmental and biodiversity sciences. The PANGAEA web submission form provides an interactive walk-through in the style of an “assistant”. Users can walk forward and backward to collect information. Once the submission is finished by the user the form creates a ticket in PANGAEA’s issue tracker. Submitters must register or use their existing ORCID ID (https://orcid.org/) for authentication. All subsequent communication between data editors and data providers is done via the issue tracking system, thus documenting each data submission and correspondence. Each submitter must accept the terms of use (https://pangaea.de/about/terms.php) and privacy policy (https://www.pangaea.de/about/privacypolicy.php). Treatment of sensible or restricted data is negotiated in direct communication with data providers.

### Review

Submitted data and metadata enter the editorial system for further inspection and review by PANGAEAs expert editors. This review ensures completeness, correctness, quality and interoperability (machine readability) of metadata and data and is labelled as “Enhanced curation”. Adaptations of metadata and data to standards are negotiated with the data providers. However, the users are responsible for the correctness of data and metadata. This is verified by the compulsory approval by the submitter before publication.

### Archiving

Data and metadata are imported into a relational database, which keeps the structure and semantics for each data entity. The data model of PANGAEA and the functionality of the editorial system enforce that each data set has sufficient and consistent metadata.

Not all of PANGAEA’s data is provided in tabular form. Some datasets are only available in compact, community specific binary formats like NetCDF or encoded as videos or static images. A list of formats accepted by PANGAEA is given in the Wiki (https://wiki.pangaea.de/wiki/Format). Raw data is not accepted by PANGAEA. Raw data in this context refers to unprocessed data without metadata (level 0). Raw data might be accepted if at least a minimum set of metadata is provided (level 1) to complement the raw data. A detailed description of the processing levels is given in https://wiki.pangaea.de/wiki/Processing_levels. Exceptions might occur for legacy data. No guarantees are given for long-term usability of those datasets.

### Publication

Each dataset is associated with a universally unique Digital Object Identifier (DOI) according to standards of DataCite. The PANGAEA team recommends citing these data publications in analogy to scholarly manuscripts. On the website the recommended citation is shown. The citation can be downloaded in different formats for further processing in reference managers. The data publication can be used “standalone” or in combination with a scientific article, e.g. 10.1594/PANGAEA.914629^6^.

Access to data is granted on the website or programmatically via the metadata catalogue (https://ws.pangaea.de). Data sets are always delivered as complete entities with metadata included. Principal investigators/authors as well as any institution or laboratory/facilities/methodologies/events contributing to the production or processing of data are recorded in the metadata (provenance).

PANGAEA uses Creative Commons (CC) licences (https://creativecommons.org/). Metadata are published under the CC Zero (CC0) and data is published under CC-BY, ensuring that authors of the data are cited. Exceptions will only be made in case of non-public funding (e.g., industry funding) with valid contracts in place. Information with recommendations to choose a suitable CC licence for data submitters can be found in the Wiki (https://wiki.pangaea.de/wiki/License). Data ownership/copyright stays with the original submitter (no copyright transfer). A minor part of the data ( < 1%) is under moratorium, mostly data from ongoing scientific projects. Protection is applied on individual and group level; metadata are always public. Protection of data is usually restricted for a time of maximum two years after submission. PANGAEA publishes around 10,000 datasets per year.

### Interoperability

PANGAEA offers a variety of ways to discover data: 1. on the website, via the PANGAEA search engine allowing for full text and faceted searches, 2. via Google Search and Google Dataset Search, 3. via numerous portals harvesting PANGAEA metadata, including the GEO data portal, INSPIRE, IODP, ICSU-WDS, PubMed Central, OpenAIRE, Scopus, Dimensions AI, DataCite, DataONE, GBIF, EMODnet, GFBio, etc. Data sets are cross-linked with literature via Scholix^7^ and with genomic data using accession numbers.

Currently, all data and metadata can be downloaded in a standard format as tab delimited text; data collections can be packed in zip folders. Formats for binary data conform to community standards. Programmatically, PANGAEA offers a wide range of web services (SOAP/REST). This includes OAI-PMH for metadata harvesting. The API allows retrieval of any set of numerical and textual data. All PANGAEA datasets also ship with schema.org/dataset metadata.

### Data warehouse

PANGAEA’s data warehouse (DWH, https://wiki.pangaea.de/wiki/Data_warehouse) is built on top of PANGAEAs entire data holding, enabling a range of meta-study and data science applications. The DWH allows spatially and chronologically confined aggregations of data on parameter level without having to compile corresponding aggregations from multiple (possibly hundreds of) individual studies, while ensuring transparency with respect to the provenance and specific properties of the underlying studies. A list of citations and DOI names is included in every export of data via the DWH, thus facilitating validation and citation. And finally, the DWH offers additional options for harmonisation like calculating daily/monthly/yearly averages and standard deviations.

### Communication and dissemination

In case of questions and support the PANGAEA team can be reached in business times via the contact form on the website (www.pangaea.de/contact/) which is connected to the ticket system. All exchange with the data providers is channelled through the ticket system to keep a full record of the information flow. The Help Page (https://wiki.pangaea.de/) provides comprehensive information on the background of PANGAEA as well as data submission (e.g., templates) and retrieval. Furthermore, a video tutorial showing the submission process of PANGAEA is available (www.pangaea.de/submit/).

To provide guidance for researchers to find appropriate repositories for their specific data output PANGAEA is registered in Re3Data^8^, Fairsharing^9^, RIsources (https://risources.dfg.de/detail/RI_00121_en.html) and the EOSC Portal (https://marketplace.eosc-portal.eu/datasources/eosc.awi_bremerhaven.2882af227241cb956c28fe321a70dfb2).

As a further communication channel, a Twitter account (@PANGAEAdataPubl) was started in 2019. Additionally, datasets can be rated via social networks including altmetric, e.g. 10.1594/PANGAEA.937204^10^.

### Usage statistics

**Fig. 2:**
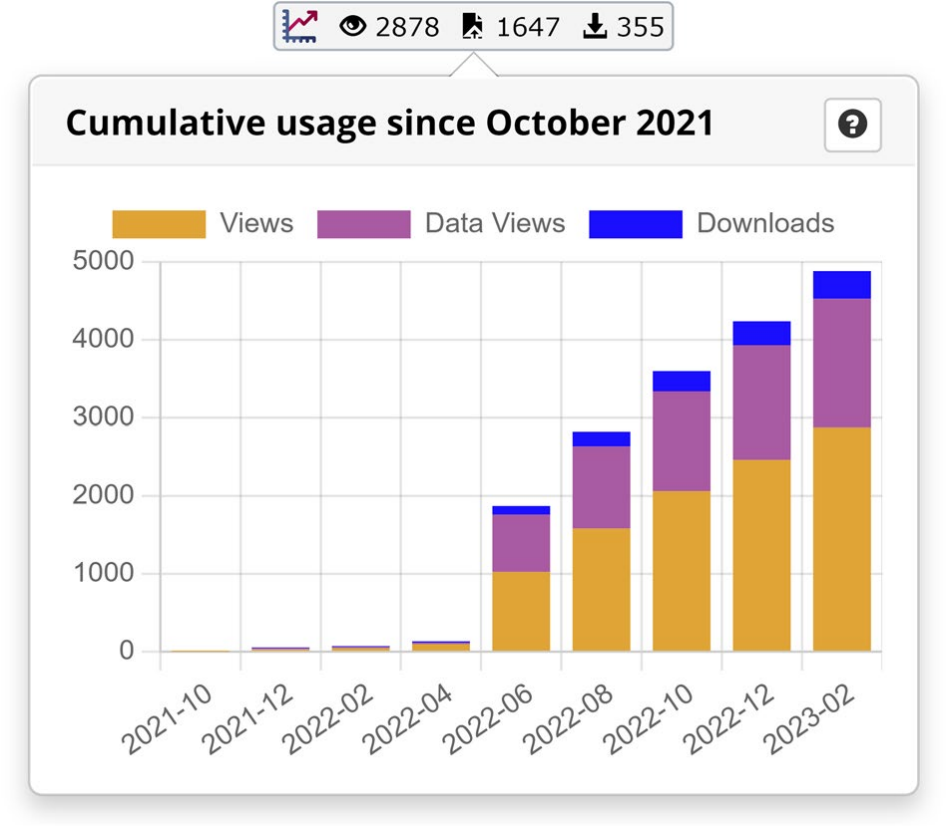
Example of a usage statistic for 10.1594/PANGAEA.937574^20^. Details on usage statistics can be found in the Wiki.

In 2022 data usage statistics for each dataset were introduced as frequently requested by the community. On the webpages of published datasets, PANGAEA offers an aggregated statistical evaluation of past interactions with related content (Fig. 2). The aggregations are generated under rigorous data protection considerations in accordance with the European Data Protection Regulation (GDPR). It does not include any personal data in the process, thus strictly preserving the anonymity of our users.

As of December 2022, PANGAEA has 19,430 registered users, with 8564 users linked to their ORCID ID (44%). During the last 12 months, 2447 new users registered with 1450 ORCID IDs (59%).

From April 2012 until December 2022 PANGAEAs landing pages of the datasets have been visited 4,2 Mio times. The corresponding data have been downloaded 657,516 times. In 2022 PANGAEAs landing pages of the datasets have been visited 551,723 and the corresponding data has been downloaded 114,975 times (https://wiki.pangaea.de/wiki/Data_Usage_Statistics).

## Discussion

### FAIRness

As pointed out in the Final Report of the European Commission on the European Research Data Landscape certain FAIR practices are being adopted, and researchers are motivated by the ideals of Open Science but obstacles still remain to making data FAIR and repositories FAIR ready^11^. Recalling an increasingly data-intensive science, and the context of pioneering infrastructure developments such as the European Open Science Cloud (https://eosc-portal.eu/) and the German National Research Data Infrastructure (NFDI)^12^ interoperability of data by machines is key. Only machine to machine communication enables an algorithm or service making decisions regarding data that it has not encountered before^3^. This automatic exchange and interpretation of the digital content is an indispensable asset in the value chain of scientific research, and an important component of the return-of-invest of publicly funded research.

PANGAEA applies a whole range of measures that render its data FAIR ready. A major asset compared to other repositories is the quality assurance of data and metadata provided by extensive direct communication of the editors with the authors. This standardizes the submissions across disciplines and therefore enhances the scientific value as well as the (re)-usability of the deposited data.

An essential feature of this process is a sufficiently precise and semantically unambiguous description and contextualisation of the data by metadata down to the level of the observables and units. PANGAEA links each observation with terms from controlled and internationally recognized vocabularies and ontologies as a measure of standardization. Examples are WORMS^13^, ITIS (www.itis.gov), ChEBI^14^, ENVO^15^, PATO (http://obofoundry.org/ontology/pato.html) and the NERC vocabularies^16^ (for details see methods section Standards/Terminologies/Ontologies). Units are harmonized (e.g., according to SI) and, if necessary, decimal places are checked for meaningfulness. This semantic unambiguity is the foundation of machine-interoperability of data and metadata. With appropriate interfaces, like those offered by the mark-up of the discovery standard schema.org (https://schema.org/), PANGAEA already fulfils the principal technical and semantic prerequisites for integration in a federated data space enabling data-driven science on larger scales.

PANGAEA’s high level of FAIRness is reached by the observation variable-level relationalization and harmonization applied to all tabular data that conform to PANGAEA’s data model. In this context relationalization means that all submitted data and metadata is organized in predefined relationships. The data is stored in tables (or “relationships”) with columns and rows. This simplifies understanding how the different data structures relate to each other. Relationships are a logical connection between different tables, established based on interactions between those tables. In the editorial process the editors evaluate, categorize and harmonise the data and metadata to fill the appropriate tables. Relationalization allows to break up the data containers, compiled to answer a specific scientific question, in a particular study, to make them interoperable and re-usable as independent variables in broader contexts. This is a centerpiece of the editorial processing and rather uncommon across earth and environmental science data publishers.

The measures undertaken by PANGAEA have been recently evaluated by the European Commission in their European Research Data Landscape report^11^ using the F-UJI FAIR assessment tool^17^. It turned out that according to this report, PANGAEA, reached the highest FAIR scores for its datasets among 31 investigated European data archives.

### PANGAEA in the course of time

Over the last decades PANGAEA has mastered to cope with the changing landscape on how the scientific community deals with research data. PANGAEA has grown in this time from a specific sediment core database to a domain specific long-term repository and data publisher for earth and environmental data. Landmarks were the introduction of the relational database model and associated with this the machine readability followed by the harvesting of its content by third-party portals. The introduction of the ticket system enhanced the transparency and documentation of the data submission and editorial process until publication. The new web-based submission system together with the new webpage improved the user experience in providing and retrieving data from PANGAEA. Along this line best practice manuals and templates as well as regular community workshops are now provided to support the authors in the preparation of their data for publication. PANGAEA’s early adoption of DOIs and the consequent advocating for data publications being an asset of its own in the data life cycle, was on the forefront of the open access, open data and finally the FAIR data movement. The introduction of usage statistics for each dataset provides tangible feedback for each author on the re-usage of the data. A good mixture of visionary technical developments in combination with reliability of the services and their instant critical evaluation by the users has brought PANGAEA through sometimes rough times.

For several years, PANGAEA has witnessed a quantitative as well as a significant increase in the complexity of data submissions. The assessment of the validity and plausibility of the data submissions, in addition to the other editorial tasks described earlier, represents a major and growing challenge. This has an impact on the time required to process a data submission. PANGAEA meets these challenges with an instant review and further development of its workflows and standards aimed at increasing the efficiency of the editorial processing. As a direct consequence PANGAEA invites data intensive groups and institutes worldwide to become part of its front-office/back-office model to make use of our professional environment for data archiving and publication.

## Methods

### Technical infrastructure and security

**Fig. 3:**
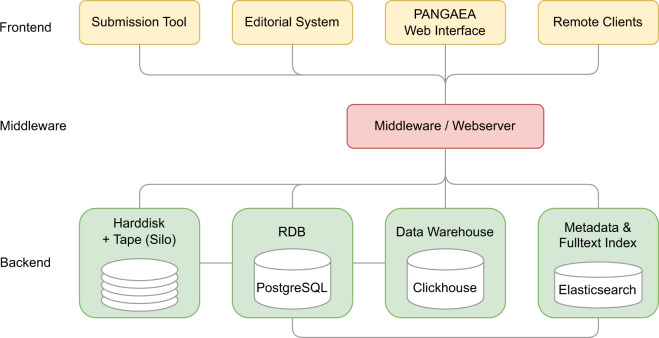
Simplified technical architecture of PANGAEA.

The technical architecture of PANGAEA follows a three-tiered client/server architecture with several clients and middleware components controlling the information flow and quality (Fig. 3). A RDBMS (PostgreSQL) is used for information storage. For better performance high volume and binary data are stored in consistent formats on hard disk arrays and tape archives. Fast access to aggregated data is ensured by a data warehouse, mirroring the data inventory. All public interfaces to the information system are standards compliant (W3C, ISO, OGC) and are based on web services (mostly SOAP, REST) including a map supported (Google Earth, Google Maps) search engine (Elasticsearch). Metadata are dynamically marshalled from the RDBMS to a PANGAEA specific metadata format, stored in Elasticsearch, and transformed (XSLT, XML to JSON transform) by the frontend to various content standards (JSON-LD according to schema.org, DataCite XML, Dublin Core XML, but also community specific ones like ISO19115/ISO19139, DIF, Darwin Core). They are disseminated via OAI-PMH, HTTP content negotiation (based on HTTP standards and technical FAIR recommendations), or other protocols.

All hard- and software services are hosted by the data and computing centre of the AWI. Most backend/middleware systems and all front-end web servers/search engines are running on virtual machines (VMware), supplying sufficient capacity and performance, as well as high availability due to virtualization. Machines are operated with Linux (Ubuntu, CentOS). The central archival storage system can keep up to 60 PB of data in two robotic tape archives (SpectraLogic TFinity ExaScale Tape Libraries). The backup facilities work on high capacity LTO-tape drives and media. PANGAEA related infrastructure and data is mirrored in two different buildings, with a third one under construction. Proper functioning of PANGAEA related systems, applications, services, and processes is ensured by usage of a professional monitoring software for the IT infrastructure.

Security of the technical infrastructure is further ensured by 1. the professional architecture and design of soft- and hardware systems (e.g., usage of asymmetric key infrastructures, passwords of data curators, system administrators, and front-end users must meet a minimum length) 2. short-term security patches for soft- and hardware (specific channels), 3. monitoring tools for hardware, firewall, software, services, performance, and attacks, and 4. regular training for technical staff, professional employment of security programs (virus scanners, firewalls, encryption programs, spam filters). Non-technical and technical staff get regular advice with specific focus on security issues like phishing, data privacy and GDPR. The host institutions guarantee that the data and metadata are available for at least 10 more years after the formal decommissioning of PANGAEA.

### Data model

**Fig. 4:**
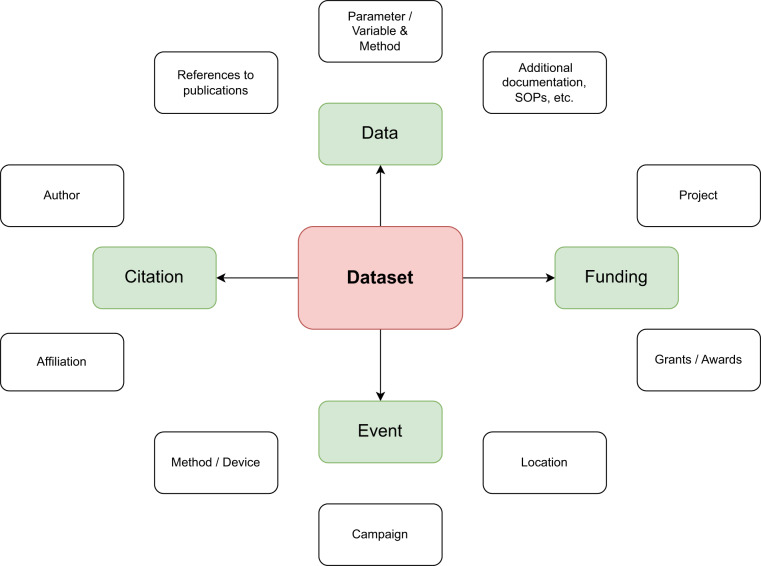
Simplified data model of PANGAEA.

The challenge of managing the heterogeneous and dynamic bio- and geosciences data was met in PANGAEA through a flexible data model. It reflects the data processing steps in the environmental science domains and can handle any relevant analytical data (Fig. 4). The system uses a normalized relational structure for data and metadata. Ingest and re-compilation of complex data sets for display or download is achieved through several middleware components—using the original data matrix configuration, stored during ingest as part of each data set.

### Data submission and editorial

The submission including all data files are stored in a special staging area accessible only by editors and submitters. Metadata from the form is collected into a JSON file and attached. The submission ticket is queued, and the user receives an e-mail with status information. At this point, no dataset and DOI is created.

During the editorial processing the following metadata is collected: Author/contributor names, their ORCID ID and affiliations with ROR identifier; title of the dataset; date of dataset publication; DOI name. Funding information (including projects and grant numbers). Event information (https://wiki.pangaea.de/wiki/Event) with detailed information when and where data was taken, including information about methods/devices. Links (including DOI names or other persistent identifiers) to related additional documentation (scientific articles/papers). If such documentation is not stored in external repositories (e.g., in libraries, publishers) and isn’t reachable by persistent identifier, PANGAEA stores a copy of the PDF file.

The relational database underlying the editorial system ensures that supplied metadata is consistent with the existing information inventory. Numerical data are checked for outliers, range and precision of values, and correct geocoding (https://wiki.pangaea.de/wiki/Geocode, deviating values are flagged). Nominal data are checked for consistency with scales supplied. Binary data are tested with customary software. Some high-volume data products like images are furnished with thumbnail information (previews).

Data objects in PANGAEA are stored as “data series” (a series of data points in numerical, date/time, string, or binary form). Each data entry in a data series refers to metadata about the object: type of data point (numeric, date, string, binary file), responsible scientist (PI), methodology, for binary files also checksums and file size, absolute location in bucket store, for numerical data also format information like significant digits.

Each data set is assigned a DOI and is minted at DataCite. The submission and archiving workflow is documented in the PANGAEA Wiki (https://wiki.pangaea.de/wiki/Data_submission#Data_publication_workflow).

### Data curation

PANGAEA currently supports two curation levels: “basic curation” which curates only metadata and “enhanced curation” which additionally curates data. This follows the Core Trust Seal Curation Levels “enhanced curation” and “data-level curation”, respectively. The PANGAEA “basic curation” comprises checks for completeness, readability and plausibility of metadata and data, as well as for compliance to PANGAEAs standards in terms of metadata quality and documentation. Data files are not entered into the RDBMS system but stored as static/binary files (e.g., shape files, NetCDF, Images).

With PANGAEAs “enhanced curation” metadata and data are harmonized as well as standardized and documentation is further enhanced. Data values are usually incorporated into PANGAEA’s relational database. Extended metadata (e.g., parameters, methods) are assigned to all data, even data themself are stored as files in community specific formats. A detailed description of the curation levels are provided in the Wiki (https://wiki.pangaea.de/wiki/Curation_levels).

### Standards/Terminologies/Ontologies

PANGAEA’s metadata format is modelled to be compliant with global standards like schema.org and DataCite metadata as well as community-based standards like ISO19115, ABCD, or Darwin Core. PANGAEA is requesting and checking ORCID IDs for all authors (registration includes request of an ORCID ID). Nevertheless, the identity of depositors is currently limited to checking institutional affiliations and mail addresses manually. Data entities and their metadata are fully documented using a detailed metadata schema (https://wiki.pangaea.de/wiki/PANGAEA_XML_schema). Usage of the terminologies (like ChEBI^14^, EnvO^15^, WoRMS^13^, ITIS or QUDT (www.qudt.org)) built into the editorial system further contribute to the harmonization of data and metadata. A terminology catalogue (TC) was embedded into the system, with interfaces to manually work on terminologies^2^.

### Workflows and templates

Archiving of data in PANGAEA follows defined workflows, which comprise technical structures, tools, and workflows as well as the organization of data and metadata by the editorial staff affiliated with PANGAEA. Recently, a series of best practice manuals and templates for different scientific domains have been introduced to best support users in submitting their data. The collection of manuals and templates can be found in the Wiki (https://wiki.pangaea.de/wiki/Best_practice_manuals_and_templates).

### Interoperability and services

Derived from the comprehensive PANGAEA metadata schema, a series of XSLT-based transformations are provided via an OAI-PMH endpoint, including standards like Dublin Core, ISO19115/19139, DataCite v3 and v4, DIF. The dataset landing pages that are resolved by the respective DOI names, are complemented with tags and rich schema.org mark-up ensuring machine-actionability. PANGAEAs flexible metadata framework PanFMP (https://www.panfmp.org/) ensures compliance with future adaptations, extensions and demands.

Metadata records in schema.org (in JSON-LD) and the other XML-based serializations are directly accessible via standardized HTTP content negotiation procedures.

Community specific formats on subsets of PANGAEA’s data holdings are available through add-on services provided on demand (e.g., metadata provided in ABCD or Darwin Core-A for biological data). Those metadata formats often have different granularity than PANGAEA dataset entities.

Direct access to data files is provided via HTTP content negotiation and REST APIs (https://ws.pangaea.de/). For scripting environments, clients for popular programming languages are available. *Python:* “pangaeapy”^18^, developed by PANGAEA / *R:* “pangaear”^19^, developed by the community. Both tools load and transform PANGAEA metadata and data into native R and Python data structures such as dataframes. This facilitates the scientific use of data in data analysis environments such as Jupyter notebooks or virtual research environments (VRE) that support these languages.

The integration of multiple internationally endorsed persistent identifier systems (PIDs) at PANGAEA, such as the Open Researcher and Contributor ID (ORCID) for the unambiguous identification of individual researchers, the Research Organization Registry (ROR, https://ror.org/) for institutional affiliation, and PIDs for projects (e.g. CORDIS, https://cordis.europa.eu/projects/en) and research funders (e.g. Crossref Funder registry, https://www.crossref.org/services/funder-registry/), as well as community-specific PIDs such as the International Generic Sample Number (IGSN, https://www.igsn.org/) and the World Register of Marine Species (WoRMS)^13^ and the Integrated Taxonomic Information System (ITIS) for organisms, complement the range of interconnectivity options for individual scientific resources and information.

### PANGAEA’s network

On the national level PANGAEA is part of the German Federation for Biological Data - GFBio (https://www.gfbio.org/), the German Network for Bioinformatics Infrastructure – de.NBI (https://www.denbi.de/) the National Research Data Infrastructure - NFDI (https://www.nfdi.de, www.nfdi4biodiversity.org, www.nfdi4earth.de) and the cloud initiative GAIA-X (https://www.gaia-x.eu/). On the European level PANGAEA is or has participated in long-term infrastructure projects e.g., ENVRI+, EMSO, ELIXIR. In addition, PANGAEA is active in the Research Data Alliance (RDA) and the European Open Science Cloud (EOSC) thus learning from other organizations and profiting from the overall development in research data infrastructures. As a funded partner, PANGAEA has supplied data management services for numerous national and international projects (https://www.pangaea.de/projects) leading to intensive collaborations with scientists. At an informal level PANGAEA staff keeps linkages with many scientists within the host institutions and worldwide to discuss and give advice for handling new data types, data policies, methodologies, and the processing of data.

### Managing the technology over the years

Starting as a core repository database (“SEDAT/SEDAN”) in 1987, PANGAEA was re-conceived as a generic, extensible system with a 3-tier architecture in 1992. The backend with a highly normalized relational database (RDBMS) allowed straightforward adaptations to structural extensions and new data content while keeping the number of tables low. Thus, it was possible to extend the scope of PANGAEA from initially relatively specialized sediment core data to nearly all types of empirical data, while meeting the ever-increasing general requirements for data and data description complexity. Furthermore, all components within the architecture, including the frontends, are well encapsulated with the effect that individual components have long half-lives and replacement of components requires only minor adjustments in other components. In addition, components were consistently replaced whenever changes or the use of new technologies resulted in a gain in functionality and/or a simplification of the system. For example, it was possible to replace the original database engine (Sybase ASE) by open-source software (PostgreSQL) in 2018 with a downtime of only a few minutes. Another decisive criterion for success is that the expertise for the technical operation and further development of the system is located within the PANGAEA team.

## Data Availability

Data and metadata in PANGAEA is freely available via the website and by programmatic access. The licence for metadata is CC0 and for data CC-BY.

## Code availability

The code supporting the users with data retrieval and submission is freely available at https://github.com/pangaea-data-publisher. PANGAEA as a repository does not generate, test, or process data and metadata, therefore no custom code has been used.
